# Caregiver’s Burden of the Patients With Traumatic Brain Injury

**DOI:** 10.7759/cureus.1590

**Published:** 2017-08-21

**Authors:** Anam Qadeer, Usama Khalid, Mahwish Amin, Sajeela Murtaza, Muhammad F Khaliq, Maria Shoaib

**Affiliations:** 1 Jinnah Medical and Dental College; 2 Department of Surgery, Shifa International Hospital; 3 Hamdard University, Hamdard university hospital; 4 Charleston Area Medical Center, West Virginia University - Charleston Division; 5 Department of Medicine, Dow Medical College, Karachi, Pakistan

**Keywords:** traumatic brain injury, caregiver stress, caregiver psychological interventions, caregiver ethnicity, caregiver social support.

## Abstract

Insufficient attention towards caregivers has resulted in the emergence of psychological and health complaints. Affliction tethers more towards spouses as compared to parents and females as compared to males. The role of sibling care givers was found to be no different from parents or spouses. Marital relationships were found to suffer the most, with the caregiver leaving the traumatic brain injury (TBI) patient in his time of need. The Brief Symptom Inventory (BSI) and family assessment device (FAD) predicted a correlation between patient variables and caregiver discontent. The Blacks/Hispanics proved to cope better with stress and their caregiver roles as compared to Whites. Time elapsed since the injury was found to relieve distress, while the surprising severity of the injury has no recorded impact. Social support or rather a lack of it has been seen to have an impact on family homeostasis, which can further be deteriorated by substance abuse by the patient. The therapeutic intervention found to be most advantageous was the D'Zurilla and Nezu social problem-solving model. Current evidence suggests that emphasis should be given on proper education and encouragement of caregivers before discharge of TBI patients from hospital to reduce the incidence of stressors. Additionally, counseling sessions should be led by professionally led support groups for dealing with psychological symptoms and peer-led group to eliminate social insecurities of caregivers.

## Introduction and background

Traumatic Brain Injury (TBI) is a non-degenerative, acquired insult to the brain secondary to any applied external mechanical force [1]. In the previous era, high mortality rates were inevitable, but innovations in the field of acute trauma care have ensured a significant reduction in the mortality rates, but unfortunately, an increase in morbidity with better survival leaves patients with multiple impairments [2]. Usually, cases of TBI are found to be accidental in nature and have deleterious effects on the mental, cognitive, behavioral and bodily functioning of the suffering individual. The patients with TBI are often impaired with short term or persistent motor and cognitive deficits and require dynamic 24hr nursing care. The major responsibility of which is placed on the shoulders of a family member who assumes the role of a primary caregiver [3]. According to popular opinion, sympathies usually lie with the patients' of traumatic brain injury. Their needs and efforts for rehabilitation are given utmost priority, however, the needs of the caregiver who has an essential role in the care of the patient with TBI are often overshadowed and ignored resulting in elevated levels of caregiver’s psychosocial burden. According to the Lazarus theory, stress is an attribute of the impact of the environment on the individual. It can further be explained as a stimulus which is provoked when the individual feels endangered because they perceive their abilities to overcome the stress to be unreliable [4]. Current studies have outlined and emphasized upon the dynamics responsible for increasing or alleviating; mental, psychological, behavioral and emotional stress in caregivers and what therapeutic approaches have proved to be beneficial for the well-being of these caregivers. This review is undertaken to help skilled personnel in the field of healthcare acknowledge and understand what challenges caregivers tackle on a daily basis, in the face of TBI.

## Review

Issues faced by singular caregiver relatives and family units

A systematic review exploring issues centered on predicaments faced by caregivers suggests that the psychological problems predominately found in caregivers include; pressure, burden, anxiety and clinical depression. Spouses were found to be more prone to be overburdened by these psychological crises as compared to biological parents, as parents are more motivated to overtake a ‘caring and providing role'. Predominant concerns of the caregivers were complaints of less personal time. Gender of the caregiver was found to have an association with the emotions felt to vent out stress; females documented psychological depression and anxiety, while on the contrary males experienced exhaustion and agitation [3]. Sixty-two families were opted for, in another large-scale study, which aimed to explore the patient variables and to predict the emerging relationship between each variable and caregiver distress/ functioning of the family. The tools used were; Brief Symptom Inventory (BSI); an 18 point self-reported scale to determine caregiver stress and family assessment device (FAD) to determine family functioning. Regression analyses demonstrated that the severity of injury had no effect on BSI scores for measuring caregiver stress, the time elapsed since injury had a considerable impact on FAD scores (p <0.05), the increasing number of neurobehavioral problems in the patient when check listed in the neurobehavioral scale down regulated the BSI scores in the caregivers most effectively. Among the ten neuropsychological scores, verbal impairment exhibited a positive correlation with the BSI scores and spouses had high depression scores as compared to biological parents due to spouses losing their marital partner, confidant, and child nurturing partner attributable to the damaging effects of TBI. [5].

Depression in adult sibling caregivers

**Figure 1:**
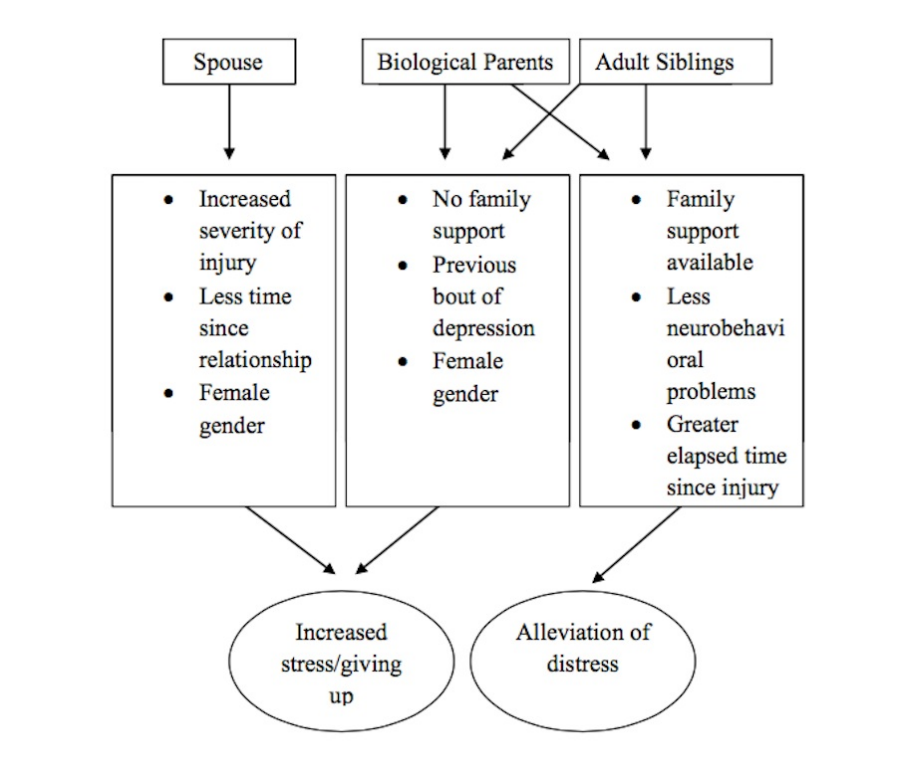
Impact of the relationship/family characteristics on caregiver stress

Siblings of the patients with TBI have been an understudied group while it is automatically assumed that they will overtake roles of responsibilities following the patient’s TBI episode. A study attempted to investigate the correlates of depression in 170 adult siblings by using Pearlin’s stress process model. The tools employed were: amount of care provided scale (ACPS) by the non-injured sibling, a 19-item cognitive-behavioral impairment scale (CBIS) of the TBI patient, subjective caregiving demand scale (SCBS) and caregiving demands scale (CDS) as perceived by the non injured sibling, family deprivation scale (FDS), future concern scales (FCS), the gain scale to measure the perceived growth of the non-injured person as a human being, frequency of family coping behaviors, the social provision scale (SPS) to measure perceived support from the society, and the center for epidemiologic studies depression (CES-D) scale to measure depression in the healthy siblings with a score of above 16 denoting clinical depression. Results demonstrated 66 of the adult siblings to have a CES-D score of higher than 16 denoting clinical depression. Depression was aggravated by the following factors; being female, a previous bout of depression, fewer responsibilities as a caregiver, and nonexistent family and social support. Higher levels of cognitive impairment of the TBI patient had no effect on depression. Similarly, lower perceived social support also aggravated depression. These findings demonstrated that the results for adult siblings were the same as those already described for other family relatives [6]. Figure 1 summarizes the impact of relationship characteristics on caregiver’s stress.

Effects of traumatic brain injury on relationships

A cohort study attempted to measure the changes occurring in close intimate relationships after an episode of TBI. Hundred and thirty-one subjects were recruited who in order to be eligible had to have a traumatic brain injury, be at least 21 years old at the time of the study, been with their partner for at least one year before TBI, and returned back to live with their partner after TBI. Further information was acquired pertaining to the severity of injury by neuropsychological assessment, the length of relationship before the TBI crises, current relationship status with the partner, and if the relationship had changed in any way. The results obtained from this showed that about 48.86% of TBI had lost their intimate partners through divorce or separation, and 6.10% of the still married respondents reported the marriage to have lost its charm. Chi-square test was conducted to find the relationship between marital stability and mature age of above 35 years old. The result (χ2=0.19 p> 0.05) denoted no relationship between the two factors. The traditional assumption that females are more likely to remain as the partner was found to be statistically insignificant ( χ2=0.02), with 45.36% of males leaving their injured partners while on the other hand, 47.1% of females partners left. The assumption that the existence of children under the age of 15 years could contribute to marital harmony and was also found to be statistically insignificant by the Chi-square-test (χ2=0). The factors contributing to relationship breakdown was where the severity of the injury, the likeliness of divorce or separation is inversely proportional to the length of relationship, and the time period of five-six years from the TBI episode to be the time when divorce or separation occurred [7].

Effects of race/ethnicity and income of caregiver

**Figure 2:**
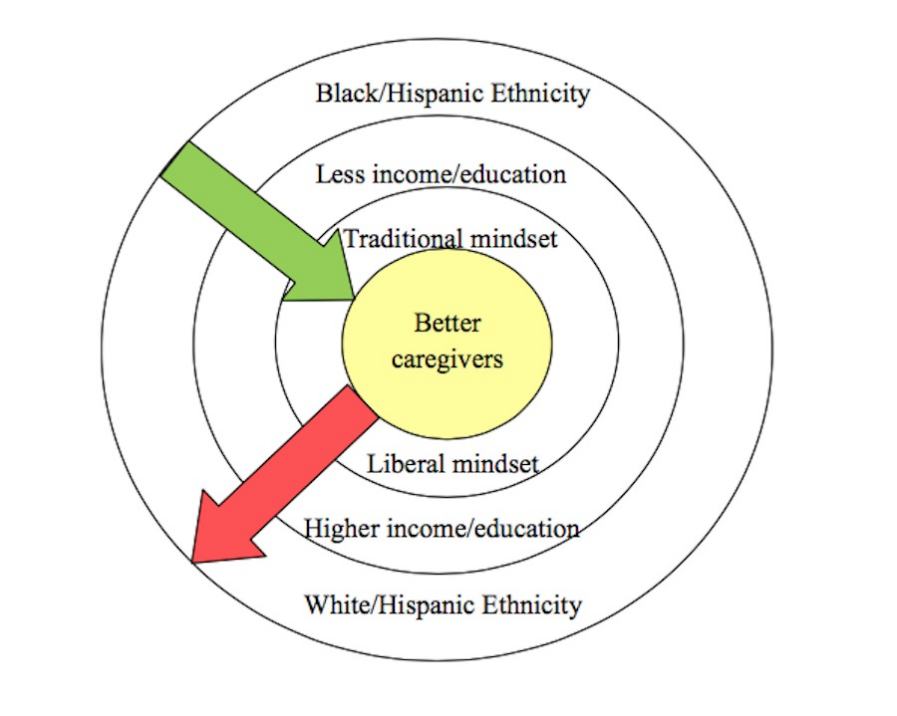
Effects of the racial/ethnicity characteristics on caregiver stress

A longitudinal study was conducted to compare emotional stress, perceived burden, and coping methods between Blacks and White caregivers. Hundred and Ninty five caregivers were enrolled out of which 75% were Whites. Socio-demographic factors measuring income, education, relationship with the patient, and Glasgow coma scale to measure the severity of injury were used. Other tools utilized were: BSI, standardized ways of the coping questionnaire (WOCQ) to identify whether the respondent used the problem-focused or emotional-focused way to cope with distress, and the modified care appraisal scale (MCAS) to assess perception of caregiving by the respondents. Results showed Blacks/Hispanics to be on the lower end of education (χ2=21.06, p < 0.01) and income (χ2=12.24, p <0.01). Race/ethnicity was found not to have any effect on levels of distress. The Blacks/Hispanics were more prone to assume a responsible caregiving role, and a more traditional mindset was found to be associated with higher levels of distress [8]. Figure 2 gives a brief idea of racial /ethnic factors in play.

Effect of perceived social support on caregivers

In addition to reporting statistically similar outcomes for aforementioned factors (severity of the injury, time since injury, the number of neurobehavioral problems, neuropsychological problems, and relation). Ergh, et al. demonstrated that social support was the strongest variable responsible for predicting family operation and demonstrated a direct linear relationship. Caregivers who received no social support were shown to have higher levels of psychological discontent while, on the other hand, while those receiving adequate support had reduced levels of stress. Alcohol and substance abuse which is a major risk factor for TBI patients is a habit that can persist even after resolution of TBI and has a congruent effect in patients with TBI and can further deteriorate their functioning and recovery, which leads to increased levels of burden on the primary and secondary caregivers [9]. A prospective cohort study predicted the outcome of the relationship between the well-being of caregivers and well-being of the patients by using post hoc test and linear regression algorithm and confirmed whether perceived social support in caregivers affected the interests of patients with TBI. Participants were 109 pairs of adults; with the pair consisting of one adult caregiver along with a patient with TBI with a Mean Glasgow Coma Scale of 8.70. The tools used in this were: BSI, satisfaction with life scale (SWLS) with higher levels indicating higher life satisfaction, disability rating scale (DRS) and neuropsychological functioning (NP Composite), and SPS. The relationship of the well-being of the pair of participants was predicted by canonical correlation. The multidimensional analysis of well-being of the patient of TBI was combined and measured by; BSI, SWLS, DRS, similarly, the multidimensional caregiver variables were embodied by; BSI, SWLS and SPS. The results demonstrated that individuals with TBI who have the following characteristics; high distress (0.83), reduced satisfaction with life (-0.38), disability (0.62) have reduced neuropsychological functioning (-0.39), which leads to lower SWLS in caregivers (=0.82), lower SPS (-0.56), higher BSI (0.37), and reduced family behavioral control (0.52). In the second step of analyses, the aim of which was to measure effects of caregiver SPS on the patient’s life satisfaction and mental health, a significant interaction (SWLS P=0.006) was observed. These findings strongly verify that the psychosocial health of the caregiver/ family has a reciprocal relationship with the individual who is under their care [10].

Alleviation of stress in caregivers after specific elapsed time points

A prospective cohort study was conducted to measure levels of psychological symptoms on caregivers of individuals with TBI after specific elapsed time-points i.e. pre-discharge, post-discharge, and three months post-discharge. Around 29 caregivers completed the caregiver strain index and depression, anxiety, and stress scales at the above-mentioned points in time. Significant levels of alleviated caregiver discontent were observed at one-month post-discharge as compared to levels at pre-discharge, and an even further reduction was observed after three months of suffering the traumatic brain episode suggesting that time elapsed injury has an inverse relation with caregiver’s perceived burden [11].

Effects of different interventions on caregivers

In contrast to the preceding studies which measured levels of stress in caregivers and factors which have a relationship with levels of stress, a recently published randomized controlled trial (RCT) evaluated an intervention in the form of a therapeutic approach known as problem-solving training program which was hypothesized to lower depression, bodily complaints and overall stressors. The caregivers were randomized into problem-solving training group (four males, 29 females) or an education-only control group (34 females). Individual with TBI in the intervention group were 26 males and seven females, whereas 24 males and 10 females were recruited for the control group. The intervention caregiver group was provided with the D'Zurilla and Nezu social problem-solving model, whereas the control group only received written educational matter. Analyses configured by the hierarchical linear models demonstrated that caregivers who received the problem-solving interventions account to a significant reductions in levels of depression, physical health, and overall social and mental comfort [12]. Sufficient recommendations to alleviate caregiver agony have not been discussed in previously existing studies. The limited research that is present clearly indicates reassurance and emotional support to be the major elements responsible for a positive outcome. In a descriptive study, it was observed that due to prompt health care now being offered, less time is spent in hospitals by the caregivers and their patients, due to which caregivers get insufficient time to finally absorb in the demands of their new role. Interviews with caregivers have revealed the reason for their dissatisfaction, which is less time given to address their concerns and proper education about medications, management, and health maintenance. Appeasement of these concerns would help caregivers step into their new role before being discharged from the hospital. An inverse correlation was found to exist between caregiver stress and receiving extensive knowledge, encouragement and facilitation [13]. In a comparative study performed to measure and then compare the efficacy of support groups led by an individual professional and those led by a group of peers, it was found that psychological symptoms tend to show better recovery when counseled by individual professionals as compared to peer-led support groups. On the other hand, support groups had their own benefit in the form of provision of the much needed social support for the caregiver. This ameliorates attached social stigmas in caregivers [14].

**Figure 3:**
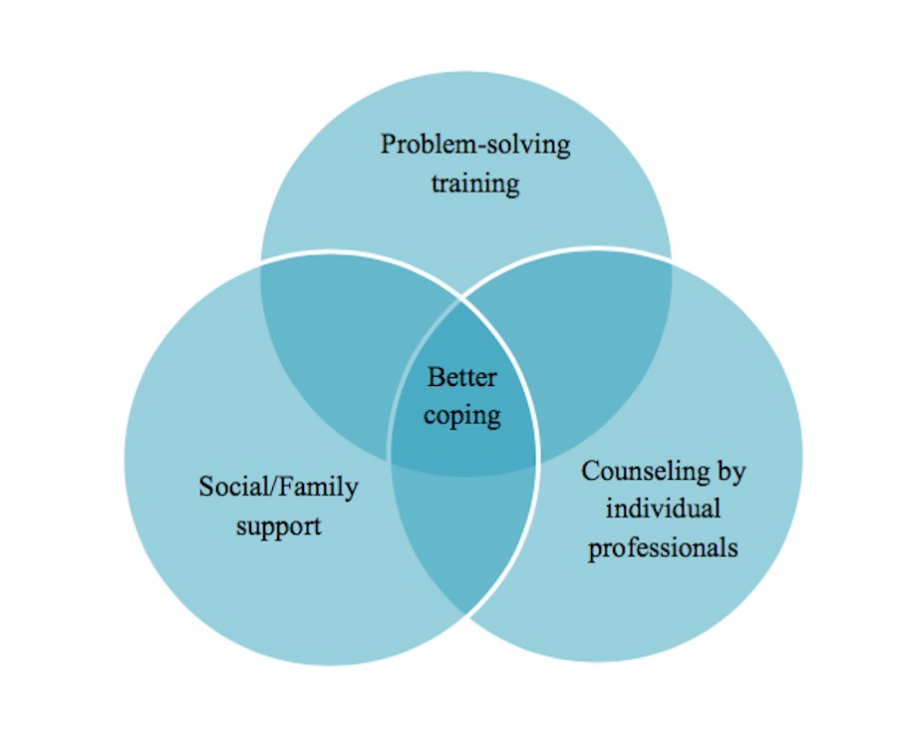
Proposed intervention strategies for alleviating caregiver stress

In the light of the above evidence, we recommend division of labor among all the family members. Although the primary caregiver, who ideally should be the most responsible and entrusted member of the family, other members should also overtake secondary roles of care giving. This secondary role should include allotting a few hours of their daily routine time to focus on the patient with TBI and completion of certain tasks of that patient. These measures would make sure that the primary caregiver has some time left for themselves, which they can spend on collecting their thoughts, coping and self-care. It is not an easy task to devote a substantial amount of one’s life span in the never-ending care of another individual, hence the caregiver deserves the utmost respect and the highest quality of care deemed necessary (Figure 3).

## Conclusions

The key conclusions by the authors in this review are factors which are affecting the varied levels of stress in caregivers. There is a definitive existence of psychological problems in overburdened caregivers, with spouses as compared to parents and females as compared to males being more on the receiving side of burden. Time since injury had a considerable impact on the family homeostasis, whereas the severity of the injury was found to have no significant impact. These findings were established to be consistent with adult non-injured siblings who had assumed the role of burden and responsibilities. Marital stability was found to suffer the most by partners separating as a result of the stress resulting from TBI, in the scenario of which females were just as to be blamed for showing less empathy as the male partners. Racial prejudice was resolved by findings being in favor of Blacks/Hispanics assuming the roles of better caregivers as compared to Whites. The amount of social support provided to the patient reflected the functioning of the family. Substance abuse in TBI individuals led to the higher caregiver stress. The strongest stress allaying factor was proved to be a therapeutic intervention in the form of problem-solving training. Recommendations include proper caregiver education before discharging the TBI patients from the hospital, which would reduce the possibilities of psychological symptoms appearing in the caregivers in the future. Stress reduction was also found to be responsive to the type of intervention given according to the specific challenges faced by the caregiver and would help them deal better with their new-found role.

## Appendices

**Acronyms**: Traumatic Brain Injury (TBI), Brief symptom Inventory (BSI), Family Assessment Device (FAD), Amount of care provided scale (ACPS), Cognitive-Behavioral impairment scale (CBIS), Subjective caregiving demand scale (SCBS), Caregiving Demands Scale (CDS), Family deprivation scale (FDS), Future concern scales (FCS), the Social provision Scale (SPS), the Center for Epidemiologic Studies Depression (CES-D) scale, Ways of coping questionnaire (WOCQ), Modified care appraisal scale (MCAS), Satisfaction with Life scale (SWLS), Disability rating scale (DRS) and Neuropsychological functioning (NP Composite).
